# RhoBTB Proteins Regulate the Hippo Pathway by Antagonizing Ubiquitination of LKB1

**DOI:** 10.1534/g3.120.401038

**Published:** 2020-02-27

**Authors:** Thanh Hung Nguyen, Adela Ralbovska, Jan-Michael Kugler

**Affiliations:** Department of Cellular and Molecular Medicine, University of Copenhagen, 2200 Copenhagen, Denmark

**Keywords:** RhoBTB, LKB1 (STK11), Hippo pathway, ubiquitination

## Abstract

The Hippo pathway regulates growth and apoptosis. We identify RhoBTB proteins as novel regulators of Hippo signaling. RhoBTB depletion in the *Drosophila* wing disc epithelium cooperated with Yki to drive hyperplasia into neoplasia. Depletion of RhoBTB2 caused elevated YAP activity in human cells. RhoBTB2 deficiency resulted in increased colony formation in assays for anchorage-independent growth. We provide evidence that RhoBTBs acts on Hippo signaling through regulation of the kinase LKB1. LKB1 protein levels were reduced upon RhoBTB2 depletion, which correlated with increased LKB1 ubiquitination. Restoring LKB1 levels rescued loss of RhoBTB in *Drosophila*. Our results suggest that RhoBTB-dependent LKB1 regulation may contribute to its tumor-suppressive function.

First discovered in *Drosophila*, the Hippo pathway has emerged as an important regulatory network controlling cell proliferation and organ size (Yu *et al.* 2015). It is further involved in tissue homeostasis and embryonic cell fate specification. Hippo signaling serves as a barrier for oncogenic transformation, highlighting the role of this pathway in regulating cancer development and progression (Zhao *et al.* 2007; Nguyen *et al.* 2014). The pathway is comprised of a core-cassette of tumor suppressors which act in a phosphorylation cascade to regulate the activity of the transcription factor YKI. Many core components of the pathway have been originally identified in *Drosophila* (Justice *et al.* 1995; T. Xu *et al.* 1995; Harvey *et al.* 2003; Udan *et al.* 2003; Harvey *et al.* 2013). A significant number of elements of the Hippo pathway are conserved between mammals and *Drosophila melanogaster* (Yu *et al.* 2015; Pfleger 2017). The mammalian pathway consists of a core cassette comprising the upstream kinases MST1/2 (Hpo in *Drosophila*) and the downstream kinases LATS1/2 (Wts in *Drosophila*). SAV1 (Sav in *Drosophila*) acts as a scaffold protein and assists in the interaction between the upstream and downstream kinases. MOB1 (Mats in *Drosophila*) plays a critical role in regulating LATS kinase activity. Activation of Hippo signaling induces the MST-dependent phosphorylation of LATS1/2 and subsequent phosphorylation of proto-oncogenes YAP and TAZ (Yki in *Drosophila*). When not phosphorylated by the Hippo core cassette, YAP/TAZ/Yki translocate to the nucleus and associate with transcriptional regulators including those of the TEAD family to induce gene expression (Lin *et al.* 2017). Consequently, Hippo pathway activity inhibits YAP/TAZ/Yki target gene expression. Dysregulation of YAP or TAZ activity has been correlated with tumorigenesis (Moroishi *et al.* 2015).

RhoBTB proteins, which include the highly similar RhoBTB1, RhoBTB2 and RhoBTB3 isoforms in human and a single RhoBTB gene in *Drosophila*, are atypical members of the Rho family. In addition to the typical GTPase domain characteristic for the Rho family, all RhoBTB proteins share a proline-rich region, two tandem broad complex, tramtrack and bric-à-brac (BTB) domains that were originally identified as motifs present in several transcription regulators in *Drosophila*, and a conserved C-terminus. Unlike the majority of other Rho family members, RhoBTB proteins do not seem to regulate the actin cytoskeleton directly (Aspenström *et al.* 2004). Instead they have been found to function through diverse molecular mechanisms (Berthold *et al.* 2008), including as substrate-specific adaptors for CUL3-based E3 ubiquitin ligase complexes. RhoBTB proteins have been implicated in mediating a variety of biological functions, including the oxidative stress response, cytoskeletal organization, apoptosis and Hedgehog signaling (Kobayashi *et al.* 2004; Zhang *et al.* 2006; Chen *et al.* 2009; Jin *et al.* 2009). Recent evidence suggests that RhoBTB proteins are deregulated in some human cancers. RhoBTB2, for example, was reported to be homozygously deleted in a large percentage of breast cancer tumors (Hamaguchi *et al.* 2002; Mao *et al.* 2009). Downregulation of RhoBTB2 was further observed in lung, bladder, bone and gastric cancer (Cho *et al.* 2009; Shi *et al.* 2008; Dong *et al.* 2012; Jin *et al.* 2013). Obtained in breast cancer cells, evidence suggests that RhoBTB2 exerts a tumor-suppressive function by inhibiting cancer cell proliferation, migration and invasiveness (Ling *et al.* 2014; Mao *et al.* 2011). Similar anti-tumorigenic properties were also found for the other RhoBTB isoforms. RhoBTB1 was found heterozygously deleted in head and neck tumors (Beder *et al.* 2005) and colon cancer (Xu *et al.* 2013). RhoBTB3 expression was greatly reduced in renal carcinoma and acts as a tumor suppressor through promoting ubiquitination and degradation of HIFα (Zhang *et al.* 2015).

Here, we provide evidence that RhoBTB proteins behave as tumor suppressors by regulating Hippo pathway activity in *Drosophila* and human cells. We show that RhoBTB2 acts via ubiquitination-dependent regulation of LKB1. Our work illustrates a novel aspect of the multifaceted molecular function of RhoBTB proteins.

## Materials And Methods

### Drosophila genetics and immunocytochemistry

The use of *Drosophila* imaginal wing discs as a model for epithelial tumor formation was previously described (Herranz *et al.* 2012; Song *et al.* 2017; Herranz & Cohen 2017). RhoBTB was a validated candidate from a genome-wide screen identifying tumor suppressors whose knockdown promoted oncogenic activity of Yki as a tumor driver in *Drosophila* (Groth *et al.* 2019 preprint).

Briefly, male flies from the KK transgenic RNAi stock library of the Vienna *Drosophila* RNAi Center (VDRC, www.vdrc.at) carrying four different inducible UAS-RNAi constructs (P{TRiP.HMC02368}attP40/CyO, P{TRiP.HMC03199}attP40, P{TRiP.HMS00411}attP, P{KK100815}VIE-260B, P{VSH330130}attP40) targeting RhoBTB were crossed to 10-15 virgins from the Yki driver stock used in the screen with the following genotype: w*, *ap*-Gal4, UAS-*GFP*/CyO; UAS-*Yki*, *tub*-Gal80^ts^**/**TM6B. Crosses were carried out at 18° and flipped to new vials after 3 days. On day 11 post-mating, larvae-containing vials were moved to a 29° incubator to induce Yki expression. Crosses were left at 29° for another 9 days and larvae were scored for size and wing disc overgrowth phenotypes on the day 20 post-crossing (induction day 10). Other fly crosses were conducted using the same protocol. All other RNAi transgenic lines including P{VSH330167}attP40 (GD), P{KK108675}VIE-260B (attP40), P{TRiP.GL00019}attP2, P{TRiP.HMS01351}attP2 (targeting LKB1) and control lines were obtained from VDRC. The UAS-LKB1 (wild type) and UAS-LKB1 (KI) fly strains (Mohseni *et al.* 2014) were a kind gift of Jongkyeong Chung.

Imaginal wing discs were dissected and processed as described (Song *et al.* 2017). Antibodies were mouse anti-MMP-1 (1:10, DSHB, 3A6B4/5H7B11/3B8D12 were mixed in equal amounts), mouse anti-Dlg (1:200, DSHB, 4F3) and rat-anti-DE-Cadherin (1:100, DSHB, DCAD2).

### Plasmids, siRNAs and shRNAs

8xGTIIC-luciferase was a gift from Stefano Piccolo (Addgene plasmid #34615). The pRL-CMV (Renilla, #E2261) was purchased from Promega (Madison, WI, USA). Smart pool siRNAs against RhoBTB2 were obtained from Dharmacon. shRNAs against RhoBTB2 were expressed from the pSuper expression vector (Brummelkamp 2002) with target sequences as listed in the Supplemental Table 1. RAS^G12V^ and LATS2 shRNA constructs were described previously (Voorhoeve *et al.* 2006).

### Quantitative reverse transcriptase PCR (qPCR)

RNA was extracted using Trizol (Invitrogen) and cDNA was prepared using the iScript cDNA synthesis kit (Biorad) with random hexamers following the manufacturer’s instructions. Specific primers used for RT-PCR are listed in Supplemental Table 1. qPCR was performed using the Solis BioDyne Firepol qPCR Master Mix and the Biorad 2X SYBR Green Master Mix.

### Luciferase assay

Luciferase assay to measure YAP/TAZ activity were performed using a dual luciferase kit (E1960, Promega) according to the manufacturer’s instructions and as previously described (Nguyen *et al.* 2017).

### Cell culture experiments

All cell lines were purchased from the ATCC and cultured under standard conditions. All cell-based assays were performed as described (Nguyen *et al.* 2017; Nguyen *et al.* 2016). YAP localization was scored using mouse-anti-YAP (sc-101199, Santa Cruz) as previously described (Nguyen *et al.* 2017). Antibodies to phospho-YAP Ser127 (Cat #4911), YAP (#4912), TAZ (#2149), LATS2 #5888), phospho-LATS1/2 (#8654), Myc-Tag (#2272), LKB1 (#3050), MARK1 (#3319) and p-MARK1-4 (#4386) were from Cell Signaling. YAP (#sc-101199) antibody used for immunohistochemical staining was obtained from Santa Cruz Biotechnology. Anti-FLAG (M2, #F3165) and RhoBTB2 (SAB1407189) were from Sigma. Anti-actin (#MAB1501) was from Millipore.

### Data availability

Strains and plasmids are available upon request. The authors affirm that all data necessary for confirming the conclusions of the article are present within the article, figures, supplementary figures and tables. Supplementary figures were uploaded to figshare. Supplemental Table S1 contains primer and shRNA sequences. Supplementary figure S1 shows phenotypes caused by additional RNAi lines used in the study. Supplementary figure S2 illustrates how YAP nuclear localization was scored. Supplementary figure S3 shows results of colony formation assays using cancer cell lines. Supplemental material available at figshare: https://doi.org/10.25387/g3.11894592.

## Results And Discussion

### Depletion of RhoBTB cooperates with Yki in a Drosophila epithelial transformation model

**Figure 1:**
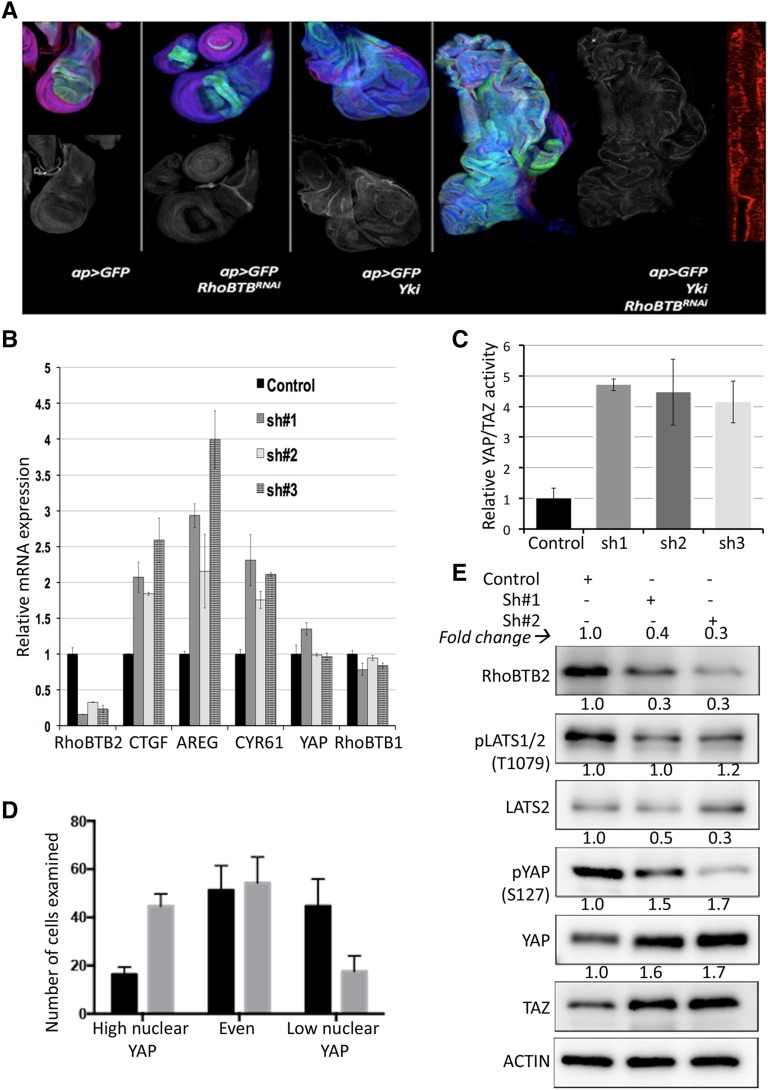
RhoBTB regulates the Hippo pathway. (*A*) Representative images showing the synergistic effect between Yki overexpression and RhoBTB depletion in a *Drosophila* transformation model. Genotypes are indicated in the figure. DAPI (blue), GFP (green), DE-Cadherin (red). MMP1 staining is shown as a separate panel (white). The rightmost image shows a representative Y-Z projection illustrating the epithelial polarity of the adjacent tumor (DE-Cadherin, red). (*B*) qPCR quantification of RhoBTB2, YAP, and YAP-targets CTGF, AREG and CYR61 in HEK293T cells treated with a control shRNA or three independent shRNAs targeting RhoBTB2. Changes in CTGF, AREG and CYR61 were significant in RhoBTB2 shRNAs-depleted cells compared to the control (ANOVA *P* < 0.001). Error bars represent mean +/− SD for three independent experiments. (*C*) Luciferase reporter assays performed in HEK293T cells treated with a control shRNA or three independent shRNAs targeting RhoBTB2. Changes in luciferase activity were significant in all shRNAs *vs.* control (ANOVA *P* < 0.0001). Error bars represent mean +/− SD for three independent experiments. (*D*) Scoring of nuclear YAP localization in BJ cells treated with a scrambled control siRNA (black bars) or a siRNA pool targeting RhoBTB2 (gray bars). Changes in number of cells exhibiting high and low nuclear YAP were significant in RhoBTB2 shRNA expressing *vs.* control cells (ANOVA *P* < 0.01). (*E*) Western blots of cellular extracts from cells treated with a control shRNA or two different shRNAs targeting RhoBTB2. Blots were probed with antibodies specific for phospho-LATS1/2 (T1079), LATS 2, phospho-YAP (S127), YAP, TAZ. ACTIN was used as a loading control. Grayscale values of inverted bands measured with ImageJ and normalized against ACTIN are shown above the corresponding bands to indicate the band intensities.

RhoBTB proteins perform a variety of molecular functions and can target proteins for proteasomal degradation (Choi *et al.* 2016). To study whether RhoBTB activity can regulate the Hippo pathway, we used a *Drosophila* epithelial tumor model (Song *et al.* 2017) which allows the spatio-temporally controlled expression of transgenes. In this system, conditional expression of Yki leads to mild hyperplasia in the imaginal wing disc epithelium (Figure 1A). RNAi-mediated RhoBTB depletion had little or no effect on its own (Figure 1A). However, co-expression of Yki with several independent RhoBTB-targeting RNAi transgenes caused formation of massively overgrown tumors in a subset of larvae (Figure 1A and Suppl. Fig. S1). On average 3-4 phenotypically noticeable larvae were observed per vial. These larvae were developmentally delayed but did not display the characteristic ‘giant larvae’ phenotype often associated with tumorigenesis in *Drosophila*. Tumor formation did not occur at high frequency, but was consistently observed in replicate experiments, independent crosses and with different RNAi lines targeting *RhoBTB*, arguing against off-target effects. The relatively low frequency of tumor-bearing larvae might be due to only partial efficiency of the transgenes targeting *RhoBTB* as well as due to competition with sibling larvae with non-inducing genotypes under crowded conditions.

Tumors did neither exhibit evident loss of polarity as assessed by several markers (DE-Cadherin, Dlg and actin), nor upregulation of Matrix Metalloprotease-1 (MMP1) (Figure 1A). Based on these observations, we decided to test whether interactions between RhoBTB activity and the Hippo pathway might be a conserved feature in human cells.

### YAP activity and YAP target expression are influenced by RhoBTB2 in human cells

YAP and TAZ are the mammalian orthologs of *Drosophila* Yki (Huang *et al.* 2005). To test whether modulation of RhoBTB activity would impact on the Hippo pathway, we utilized a luciferase reporter containing octameric TEAD binding sites in HEK293T cells. In these cells, the reporter responds sensitively to perturbations of YAP and TAZ activity (Dupont *et al.* 2011; Nguyen *et al.* 2016). HEK293T cells express high levels of RhoBTB2. Of note, HEK293T and the other cell lines used in this study expressed very low levels of RhoBTB1 mRNA as compared to RhoBTB2 and undetectable levels of RhoBTB3 (data not shown). Three independent RhoBTB2 shRNAs, which strongly reduced RhoBTB2 expression but had little or no effect on RhoBTB1 (Figure 1B), strongly induced reporter activity (Figure 1C). Consistently, several endogenous transcriptional YAP targets (CTGF, AREG an CYR61) were upregulated upon depletion of RhoBTB2, while there was no change in YAP mRNA levels (Figure 1B). Taken together, these results indicate that RhoBTB2 possibly influences YAP activity post-transcriptionally through regulating the Hippo pathway.

### YAP is post-transcriptionally regulated by RhoBTB2

Upon phosphorylation by the Hippo pathway core cassette components LATS1 and LATS2, YAP is retained in the cytoplasm and ultimately targeted for degradation (Zhao *et al.* 2010). In contrast, non-phosphorylated YAP translocates to the nucleus to activate target gene expression. Since knockdown of RhoBTB2 led to an increase of YAP/TAZ activity, we asked whether RhoBTB2 inhibition would affect the subcellular localization of YAP. We utilized a previously established fibroblast cell model expressing human TERT, p53 shRNA, p16 shRNA and RAS^G12V^ (BJ^hTert/p53kd/p16kd/RASG12V^) that permits detecting YAP translocation by immunochemical staining of YAP upon inhibition of the Hippo pathway (Nguyen *et al.* 2017). In comparison to control cells, RhoBTB2 depletion caused a statistically significant shift toward increased nuclear YAP levels (Figure 1D and Suppl. Fig. S2). We next assessed the expression and phosphorylation of Hippo proteins in the context of RhoBTB2 depletion in these cells by Western blot analysis. Knockdown of RhoBTB2 using two different shRNAs lead to an increase in the protein level of YAP and TAZ (≥ 1.fivefold increase in protein levels, which correlated with reduced phosphorylation of YAP (p-Ser127: reduced by more than 0.5 fold) (Figure 1E). LATS2 protein levels did not change or increased slightly (∼1.twofold). In contrast, we observed a more than twofold reduction of LATS phosphorylation, consistent with reduced LATS activity.

Taken together, these data are consistent with the idea that RhoBTB2 deficiency prevents the Hippo core cassette from phosphorylating YAP, which would consequently promote transcription of target genes in the nucleus. Therefore, our results suggest that RhoBTB2 regulates YAP at the post-transcriptional level through the Hippo core cassette.

### RhoBTB2 inhibits cell growth by regulating Hippo pathway activity

**Figure 2:**
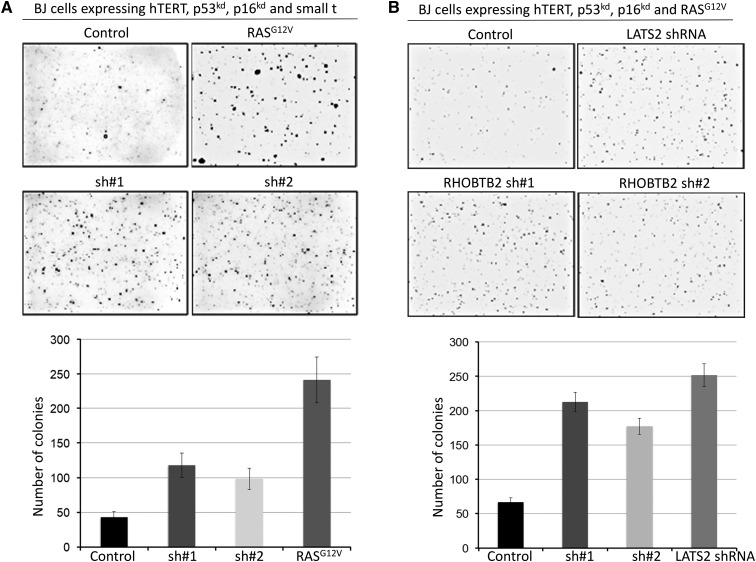
RhoBTB2-depletion enhances anchorage-independent growth in soft agar assay. (*A*, *B*) Colonies formed in soft agar by partially transformed BJ cells (genotypes indicated in the figures) treated with either a control shRNA, or shRNAs targeting RhoBTB2. Upper panels: representative images. Lower panels: Graphs showing number of colonies per image from three experiments of independently transduced cells. RAS^G12V^ and LATS2 shRNA were used as positive controls for each cell model. Changes in colony number were significant in RASG12V and all shRNA-expressing *vs.* control cells (ANOVA *P* < 0.01). Error bars represent mean +/− SD for three independent experiments.

Active YAP can promote cell proliferation and growth via transcriptional regulation of numerous target genes (Zhao *et al.* 2007; Ehmer & Sage 2016). We therefore asked whether depletion of RhoBTB2 would cause phenotypes associated with increased YAP activity. To address this question, we studied the effect of RhBTB2 suppression on colony formation in soft agar in two partially transformed human fibroblast cell models expressing human TERT, p53 shRNA, p16 shRNA and small T (BJ^hTert/p53kd/p16kd/small t^) or RAS^G12V^ (BJ^hTert/p53kd/p16kd/RASG12V^). These genetically defined cells were previously shown to be sensitive to perturbations of YAP activity, which is coordinately regulated by the Hippo and RAS pathways (Nguyen *et al.* 2014; Hong *et al.* 2014). As expected, expression of active RAS (Figure 2A) or depletion of LATS2 (Figure 2B), which induces YAP activation, caused enhanced transformation phenotypes upon inhibition of Hippo activity. Depletion of RhoBTB2 in these cell lines increased anchorage-independent growth in soft agar assays (Figure 2A and 2B). These data are consistent with the idea that RhoBTB2 controls cell growth and proliferation by modulating Hippo pathway activity.

We observed a similar growth-suppressive behavior of RhoBTB2 in several cancer cell lines. Depletion of RhoBTB2 led to an increase in colony formation in the cell lines HT-15, MDA-MB-468, HeLa, HCT116 and HT-29 (Suppl. Fig. S3). The cell lines MDA-MB-231, DU-145, A549 and H1299 did not display a change in colony numbers. Surprisingly, MCF-7 cells showed reduced cell growth when RhoBTB2 was depleted (data not shown). The role of RhoBTB2 in these cell lines remains unclear. Taken together, however, these results suggest that RhoBTB2 could be part of the machinery that limits proliferation of cancer cells, depending on the specific cellular context.

### RhoBTB2 acts on the Hippo pathway via regulation of LKB1

**Figure 3:**
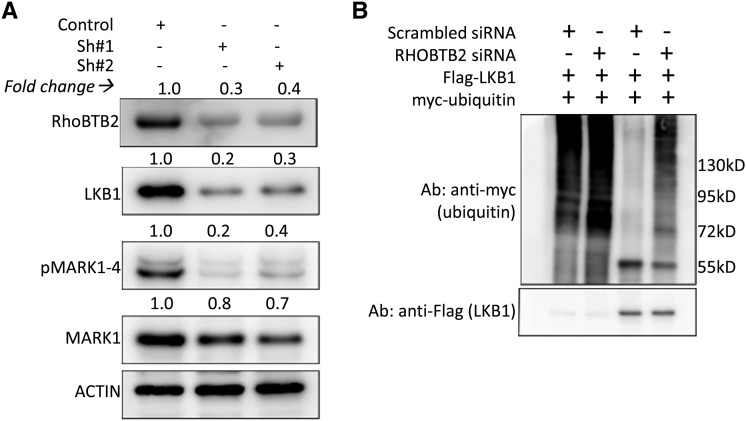
RhoBTB2 depletion affects the Hippo pathway through the LKB1/MARK axis. (*A*) Western blots of HEK293T cells transfected with a control shRNA or two independent shRNAs targeting RhoBTB2. Blots were probed for LKB1, phospho-MARK1-4 and MARK1 antibodies. ACTIN was used as a loading control. Grayscale values of inverted bands measured with ImageJ and normalized against ACTIN are shown above the corresponding bands to indicate the band intensities. (*B*) Ubiquitination assay of cells transfected with myc-Ubiquitin, FLAG-LKB1 and either a scrambled siRNA pool, or with a siRNA pool targeting RhoBTB2. Blots were probed with anti-myc and anti-FLAG.

To elucidate how RhoBTB2 regulates Hippo signaling, we searched the BioGRID database (Oughtred *et al.* 2018) for potential interaction partners. This approach identified the kinase LKB1 (also known as STK11) as a candidate RhoBTB2 interactor. LKB1 acts on the Hippo pathway via phosphorylation of MARK kinases and thereby modulates YAP activity to regulate organ growth and proliferation (Mohseni *et al.* 2014). We observed reduced LKB1 expression (≥ threefold) and MARK phosphorylation (40% reduction) in RhoBTB2-depleted cells (Figure 3A). Since LKB1 is a known substrate of the Skp2-SCF ubiquination complex that targets several Hippo components (Lee *et al.* 2015), we asked if RhoBTB2 depletion would affect LKB1 ubiquitination. Consistent with the reduced LKB1 levels, LKB1 was more extensively ubiquitinated in RhoBTB2-depleted cells than in the control (Figure 3B). These data are consistent with the idea that RhoBTB2 impacts on Hippo pathway activity through the LKB1/MARK axis.

### Resupply of LKB1 rescues tumor formation induced by RhoBTB depletion

**Figure 4:**
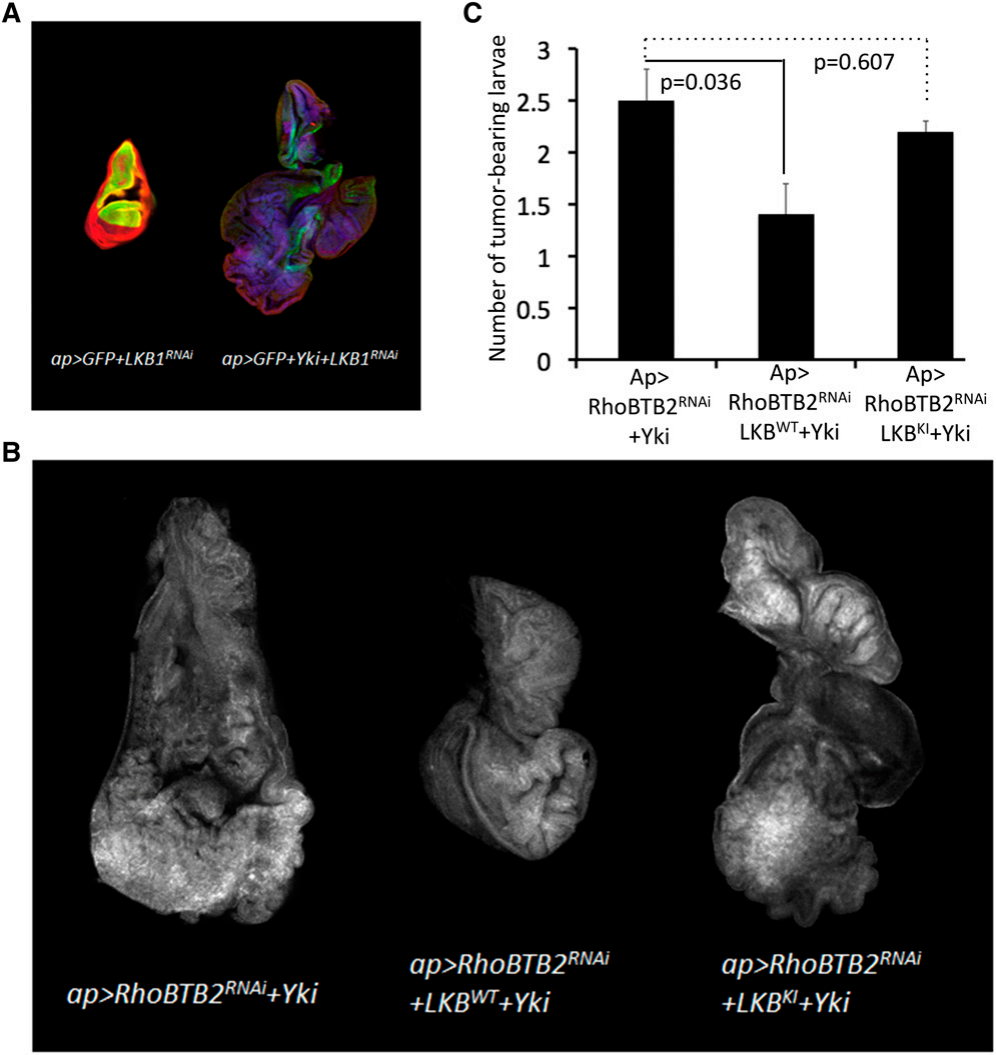
LKB1 resupply suppresses tumor formation induced by depletion of RhoBTB2. (*A*) Confocal images of wing discs showing the effect of LKB1 depletion. GFP (green), DAPI (blue) and Dlg (red). Genotypes are indicated in the figure. All images correspond to the same scale. (*B*) Representative images of wing discs showing the influence of wildtype LKB1 overexpression respectively kinase-inactive LKB1 expression on the RhoBTB2 depletion phenotype. Discs were stained for actin. Genotypes are indicated. All images were taken at the same magnification. (*C*) Graphs showing the effect of wildtype LKB1 (LKB1^WT^) overexpression respectively kinase-inactive LKB1 (LKB1^KI^) expression on the frequency of wing disc tumor formation. Data are presented as the number of tumor-bearing larvae scored per day ± SD *p*-values were determined using a non-parametric Mann-Whitney test.

Depletion of RhoBTB drives Yki-mediated hyperplasia into tumor formation (Figure 1A). To test whether a decrease in LKB1 levels produces a similar effect, we depleted LKB1 in the *Drosophila* wing disk epithelium using several independent RNAi lines. Concomitant with *Yki* overexpression, but not on its own, LKB1 depletion led to the formation of larvae bearing large tumors (Figure 4A). We did neither detect a loss of epithelial polarity nor significant changes in MMP-1 levels or occurrence of giant larvae (not shown). The phenotypical similarities between individuals depleted for RhoBTB and LKB1 strengthen the idea that RhoBTB acts on the Hippo pathway via regulation of LKB1.

The above-described results prompted us to investigate whether resupply of LKB1 could rescue tumor formation induced by RhoBTB depletion. We therefore transgenically elevated LKB1 levels in RhoBTB-depleted larvae overexpressing *Yki*. Addback of wildtype LKB1 was correlated with a lower frequency of tumor-bearing larvae while expression of a kinase-inactive form of LKB1 did not rescue tumor formation (Figure 4B and 4C). We further examined the morphology of wing disc tumors and observed phenotypic suppression in a substantial fraction of the individuals overexpressing wildtype LKB1. No suppression was obvious when overexpressing kinase-inactive LKB1. These data demonstrate that LKB1 can compensate for loss of RhoBTB function, supporting the hypothesis that RhoBTB proteins regulate Hippo signaling through mediating LKB1 turnover.

Based on our data, we cannot rule out that LKB1 affects growth independent of Yki/YAP/TAZ. However, given that RhoBTB depletion clearly affects core components of the Hippo pathway, and that LKB depletion on its own does not affect growth in the imaginal wing disk but requires elevated Yki levels to drive tumor formation, the most parsimonious model suggests that RhoBTB proteins impact on growth through the Hippo pathway by ultimately influencing Yki/YAP/TAZ abundance and activity. We cannot rule out that LKB1 acts on growth through additional mechanisms that do not impact on Yki/YAP/TAZ.

In this study, we have provided evidence that RhoBTB proteins may modulate Hippo pathway activity via regulation of LKB1. Our results indicate that RhoBTB2 plays a regulatory role in a ubiquitination cascade acting on LKB1, thereby preventing its ubiquitination and subsequent proteasomal degradation. These findings provide insights into the tumor-suppressive function of RhoBTB proteins and into the upstream signaling processes controlling the Hippo pathway.
